# Participation in the Georgia Food for Health programme and CVD risk factors: a longitudinal observational study

**DOI:** 10.1017/S1368980023001611

**Published:** 2023-11

**Authors:** Miranda Alonna Cook, Kathy Taylor, Tammy Reasoner, Sarah Moore, Katie Mooney, Cecilia Tran, Carli Barbo, Stacie Schmidt, Aryeh D Stein, Amy Webb Girard

**Affiliations:** 1 Laney Graduate School, Emory University, Atlanta, GA 30322-1007, USA; 2 Open Hand Atlanta, Atlanta, GA, USA; 3 Grady Health Systems, Atlanta, GA, USA; 4 Wholesome Wave Georgia, Atlanta, GA, USA; 5 Department of General Internal Medicine, Emory University School of Medicine, Atlanta, GA, USA; 6 Hubert Department of Global Health, Rollins School of Public Health, Emory University, Atlanta, GA, USA

**Keywords:** Food security, Nutrition intervention, Produce prescription, Health equity

## Abstract

**Objective:**

To assess the relationship between programme attendance in a produce prescription (PRx) programme and changes in cardiovascular risk factors.

**Design:**

The Georgia Food for Health (GF4H) programme provided six monthly nutrition education sessions, six weekly cooking classes and weekly produce vouchers. Participants became programme graduates attending at least 4 of the 6 of both the weekly cooking classes and monthly education sessions. We used a longitudinal, single-arm approach to estimate the association between the number of monthly programme visits attended and changes in health indicators.

**Setting:**

GF4H was implemented in partnership with a large safety-net health system in Atlanta, GA.

**Participants:**

Three hundred thirty-one participants living with or at-risk of chronic disease and food insecurity were recruited from primary care clinics. Over three years, 282 participants graduated from the programme.

**Results:**

After adjusting for programme site, year, participant sex, age, race and ethnicity, Supplemental Nutrition Assistance Program participation and household size, we estimated that each additional programme visit attended beyond four visits was associated with a 0·06 kg/m^2^ reduction in BMI (95 % CI –0·12, –0·01; *P* = 0·02), a 0·37 inch reduction in waist circumference (95 % CI –0·48, –0·27; *P* < 0·001), a 1·01 mmHg reduction in systolic blood pressure (95 % CI –1·45, –0·57; *P* < 0·001) and a 0·43 mmHg reduction in diastolic blood pressure (95 % CI –0·69, –0·17; *P* = 0·001).

**Conclusions:**

Each additional cooking and nutrition education visit attended beyond the graduation threshold was associated with modest but significant improvements in CVD risk factors, suggesting that increased engagement in educational components of a PRx programme improves health outcomes.

Suboptimal diet quality accounts for a greater population burden of morbidity and mortality from chronic diseases than tobacco, alcohol and physical activity combined^(1)^. Consumption of diets including high proportions of fruits and vegetables are associated with reduced risks of developing CVD, type 2 diabetes and cancer^(2)^. However, the majority of US adults consume less than the recommended amounts^(3)^. This is especially true for individuals facing food insecurity, the limited or uncertain ability to acquire adequate food due to insufficient money and other resources^(4,5)^.

Individuals experiencing food insecurity may employ compensatory strategies such as skipping meals, reducing portion sizes and reducing variety in their diets, which can increase the risk of development or exacerbation of diet-sensitive chronic disease^(6,7)^. A combination of physiological and behavioural responses to food insecurity and the associated stress has been offered as an additional explanation for the observed relationships between food insecurity, suboptimal diets and chronic disease^(7–9)^. This is especially salient for low-income black populations in the Southeast US who experience disproportionate chronic disease burden^(10)^. Even when controlling for socio-economic status, significant racial differences in chronic disease outcomes are evident^(11)^. Structural, institutional, interpersonal and internalised racism lead to health inequalities through social, economic and political exclusion resulting in less access to resources and greater physiological embodiment of stress, both of which lead to poorer health outcomes^(12)^.

Given the important roles of diet quality and food insecurity in chronic disease^(3,7)^, there has been a proliferation of interest in interventions incorporating Food is Medicine™ initiatives into healthcare systems to facilitate access to healthy foods for marginalised patients^(13–15)^. One such approach is a produce prescription (PRx) model, in which healthcare providers refer their patients to free or discounted healthy produce^(14,16)^.

PRx programmes use a partnership model of care that involves a referring healthcare provider and produce retailers^(17)^. Financial incentive models, including PRx programmes, are informed by the principles of operant conditioning, whereby behaviours eliciting rewards are repeated^(18,19)^. Incentives, in this case produce, may act as facilitators for healthy cooking and eating practices by increasing access and convenience of acquiring fresh produce, enabling participants to practice skills outside of class sessions and build self-efficacy. In this way, incentives act as catalysts for behaviour change and repeated engagement may become intrinsically motivating, facilitating sustained behaviour change^(20)^. Some PRx programmes incorporate group-based nutrition education and cooking sessions^(21–26)^. Nutrition education increases knowledge and awareness while hands-on cooking sessions provide skills and increase self-efficacy to engage in the behaviour^(27–29)^. These behaviours are then reinforced through educational sessions involving peer and provider support and through practice at home as facilitated by the provision of free or discounted produce^(17,20,27,30)^.

There is consistent evidence that PRx programmes increase food security and fruit and vegetable consumption^(16)^. However, few studies have reported on health outcome measures. A recent meta-analysis estimated that PRx programmes are associated with decreases in BMI of 0·6 kg/m^2^ (95 % CI 0·2, 1·1) and HbA1c of 0·8 % (95 % CI 0·1, 1·6) with no significant changes observed for blood pressure or lipid concentrations^(16)^. Among studies reporting health outcomes from participation in PRx programmes, only one used longitudinal data^(22)^ and none to our knowledge has reported on multiple years of programme implementation. Additionally, no studies to our knowledge have assessed the relationship between programme attendance and health outcomes within the context of a PRx programme. No studies evaluating health outcomes have been conducted in the Southeastern US or with predominantly black participants to our knowledge. Given the health disparities in this population^(31)^, there is a great need for research to include more black participants and other underrepresented groups. To address these needs, we assessed the relationship between programme attendance and changes in CVD risk factors in the Georgia Food for Health (GF4H) programme, a PRx programme implemented in inner-city Atlanta, Georgia with a majority black participant population.

## Methods

GF4H is a multi-partner collaboration that aims to improve food access and provide experiential nutrition and cooking education. The 6-month GF4H programme provided vouchers worth $1 per household member per day, redeemable weekly for fresh produce at retail locations throughout Atlanta. Additionally, participants received monthly group-based nutrition education and hands-on cooking classes for the first 6 weeks of the programme.

### Local context and partnership roles

Located in inner-city Atlanta, Georgia, Grady Health Systems is a safety-net hospital that served as the healthcare partner and implementation site for the programme. Grady Health Systems serves marginalised populations in Fulton and Dekalb counties who have limited or no health insurance. Data collected from the Grady Health Systems Primary Care Center suggest that the majority of patients experience poverty (90 % report annual family incomes < $20 000), multiple chronic health conditions (two-thirds have ≥ 4 chronic diseases) and demonstrate low patient activation (60 % report low knowledge and confidence to take action in self-management of health). Open Hand Atlanta is a community-based organisation that served as the cooking education partner and provided funding for produce. Wholesome Wave Georgia is a community-based organisation that provided administrative support and funding for produce. The Common Market Southeast, the East Point Farmers Market and the MARTA markets, a local food distributor and community farmers markets, respectively, provided produce and prescription redemption sites for the programme. Emory University is a research institution and served as the research and evaluation partner.

### Recruitment

Participants were referred by healthcare providers from five clinics within Grady Health Systems including three primary care clinics, a diabetes clinic and an infectious disease clinic. Eligibility requirements included a positive screen for food insecurity in the previous 12 months using a validated 2-item food insecurity screener^(32,33)^. Participants were 18 years or older, patients of the Grady Health Systems Primary Care Centers and expressed commitment to the 6-month programme^(32)^. Recruitment strategies varied somewhat by year and clinic. In 2017, clients were referred directly by their healthcare providers during clinic visits and followed up by registered dietitians for enrolment into the programme. In 2018 and 2019, participants from four of the five clinics were recruited from a pool of patients who were attending group nutrition education sessions offered at the clinics by registered dietitians. At the fifth clinic, participants were referred directly during clinic visits by their healthcare providers and followed up by registered dietitians for enrolment.

### Intervention

Over the first 6 weeks, six hands-on cooking classes were taught by a Registered Dietitian from Open Hand Atlanta using Cooking Matters™, an evidence-based curriculum^(34)^. Classes included resource management tips, with the goal of teaching participants to prepare healthy meals on a limited budget. At each weekly cooking skills class, seasonal produce was provided according to participant household size. Concurrently, participants attended monthly Eat Well, Live Well wellness courses for the duration of the 6-month GF4H programme. The education content of the Eat Well, Live Well nutrition sessions covered shopping and cooking healthfully on a budget, exercise demonstrations and gardening sessions. At each monthly Eat Well, Live Well nutrition session, vouchers were distributed worth $1 per family member per day. These were redeemable at local retail locations such as MARTA markets and farmers markets located in train stations in participants’ communities. To address common barriers to participation, the GF4H programme offered assistance with transportation, allowed participants to bring children to group sessions and offered opportunities to make up missed group sessions with one-on-one meetings with providers as needed. See Table 1 for a description of each component of the programme.

**Table 1:** Georgia Food for Health programme components

Component	Description
1. Nutrition and cooking instruction	For the first 6 weeks of the programme, participants engaged in weekly, 2-h long, hands-on nutrition and cooking instruction following the Cooking Matters™ curriculum. Topics covered included MyPlate, reading nutrition labels, knife safety, food safety, meal planning, shopping on a budget, comparing unit prices and incorporating more fruits, vegetables, whole grains and healthy fats into the diet while reducing Na, sugar and unhealthy fats in the diet. The first hour of each week included educational activities and the second hour involved collaboratively cooking and eating a healthy recipe together
2. Nutrition education	Once a month, participants engaged in a 1-h nutrition education session developed and led by a registered dietitian called ‘Eat Well, Live Well’. Topics covered included nutrition basics, portion sizes and meal planning, healthy eating during the holidays, alternative ways of eating (vegetarian, pescatarian, plant forward), exercise and urban gardening. Data collection occurred immediately prior to education sessions and healthy snacks and beverages were provided each session
3. Produce	Participants redeemed vouchers for fresh, local fruits and vegetables weekly at the health clinic for the first 6 weeks and then at partner farmer markets until the end of the programme (6 months)

### Graduation

Participants were considered graduates if they attended 4 out of 6 of both the Cooking Matters classes and Eat Well, Live Well sessions. In 2017, forty-three participants were enrolled in the programme across two cohorts and thirty-four of those participants graduated (79 %). In 2018, the programme expanded, adding additional cohorts with 115 participants enrolled. Of those, ninety-one graduated (79 %). In 2019, 173 participants were enrolled and 157 graduated (91 %).

### Measures

Surveys were administered at baseline, at the final Cooking Matters session 6 weeks later, and at the end of the programme 6 months following baseline. Surveys were self-administered by participants with evaluators present to assist with questions, verbally administer surveys as needed and check for survey completion.

Socio-demographic information collected at baseline included sex (female, male), age in years (18–29, 30–39, 40–29, 50–59 and 60+), ethnicity (Hispanic or Latino: Yes/No), race (Asian/Asian American, American Indian/Alaskan Native, Black/African American or Caribbean American, Hawaiian/Pacific Islander, White/Caucasian and Other/Multi-racial), highest level of education attained (less than high school degree, high school or GED certificate, two-year college or technical school degree, some college/technical school, but have not graduated, four-year college or technical school degree and more than four-year college degree), employment status (working full-time, working part-time, retired, not employed/homemaker, student, on disability and other), health insurance status (uninsured, insured by Medicaid, Medicare or other public insurance, insured through employer, insured through private insurance and other), annual household income (less than $25 000, $25 000–$34 999, $35 000 or greater) and household size including non-relatives living in the home.

The 2-item Hunger Vital Signs tool^(32)^ was used to determine eligibility for the programme. At enrolment, 6 weeks of participation and the end of the programme, participants completed the 6-item United States Department of Agriculture Household Food Security Survey Module^(35)^ with a 30-d recall to assess recent food security status and change over time. The 6-item module was chosen over the longer 18-item United States Department of Agriculture module for programme evaluation to avoid unduly increasing participant burden while still providing granularity of food security status beyond that of the 2-item tool used in recruitment^(35)^. Food security was categorised using the scoring guide with categories including: high or marginal food security (0–1 affirmative responses to screening questions), low food security (2–4 affirmative responses) and very low food security (5–6 affirmative responses)^(35)^.

At each monthly Eat Well, Live Well visit, clinical staff collected height, weight, blood pressure and waist circumference prior to programme education sessions. Height was collected using ScaleTronix stadiometers, weight using ScaleTronix scales, blood pressure using Omron Blood Pressure Monitor Model BP742N and waist circumference using retractable measuring tape. BMI was derived from monthly height and weight variables as weight in pounds divided by height in inches squared and multiplied by 703^(36)^.

Redeemed vouchers were collected by the individual markets at the time of redemption and returned to study staff. Household per-capita redemption was calculated as the dollar amount of vouchers redeemed divided by household size.

### Ethics

This project was deemed exempt from review by Emory University’s institutional review board, as it was considered a quality improvement project for an existing and ongoing intervention and was approved by Grady Health Systems’ Office of Research Administration. Though informed consent was not required, participants were informed of data collection procedures and informed that all data collection was voluntary, and they could choose not to participate in these procedures without affecting their ability to continue in the programme.

### Analytic sample

Participants who were enrolled but did not complete the programme (*n* 49) were excluded from the analysis as follow-up data were not available for those who did not complete the programme due to alignment of programme sessions and data collection. The mean number of visits attended among those lost to follow-up was 1·1, meaning only baseline data were available for those lost to follow-up, limiting our ability to conduct an intent-to-treat analysis. The overall graduation rate across all 3 years was 83 %, resulting in a final analytical sample of 282. We conducted an attrition analysis comparing socio-demographic, household characteristic and food security information provided at baseline for those retained and those lost to follow-up using frequencies and chi-square tests to identify significant differences between the groups.

### Statistical methods

We used descriptive analyses, including means and frequencies to characterise study participants and paired *t* tests to test the significance of change in values for continuous outcomes. We used a longitudinal, repeated measures, single-arm approach to estimate the association between the number of monthly programme visits attended and changes in BMI, weight, waist circumference, systolic blood pressure and diastolic blood pressure. In this study, we restricted the analysis to programme graduates, restricting the range of monthly visits attended to 4–6, so while the model uses all available data from visits 1–6 in estimation, the coefficients reflect the association between a one-unit increase in visits attended beyond visit 4 and outcome. We controlled for potential confounding factors by including fixed effects for programme site, year, participant sex, and age, race and ethnicity, Supplemental Nutrition Assistance Program participation, and household size and random effects for intercepts and slopes for participants and site of participation, which accounts for individual and site-level variation in outcomes at baseline and over time. Fixed effect covariates were selected using forward selection procedures and comparing Akaike information criterion and Bayesian information criterion values as indicators of model fit. We used restricted maximum likelihood to estimate the model parameters and we presented estimates with 95 % CI.

Some socio-demographic data were missing for fifty-five of the programme graduates (19·5 %). Specifically, race and ethnicity were missing for 8 (2·8 %), highest level of education attained was missing for 7 (2·5 %), health insurance status was missing for 38 (13·5 %), employment status, income or receipt of public benefits was missing for 6 (2·1 %, respectively) and household size was missing for 9 (3·2 %). Some covariate data were additionally missing: sex was missing for 4 graduates (1·4 %) and age was missing for 9 (3·2 %). Additionally, blood pressure was missing for 1 observation for 7 graduates (2·5 %), BMI was missing for 3 (1·1 %) and waist circumference was missing for 5 (1·8 %). We used multivariate imputation by chained equations method to estimate observed outcomes in the scenario of no missing data (see online Supplemental Materials)^(37)^. All analyses were conducted in STATA version 17.0^(38)^. Statistical significance was determined at *P* < 0·05.

## Results

### Participant characteristics

**Fig. 1:**
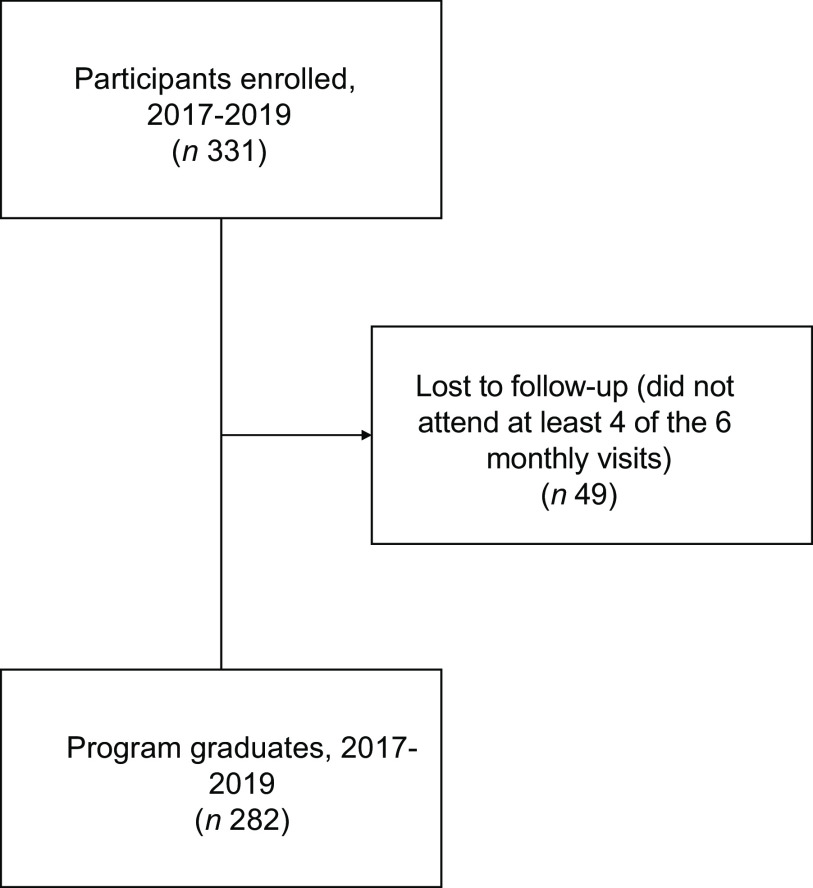
Flow chart of participants enrolled, lost to follow-up and final analytical sample of programme graduates

A flow chart displaying the number of participants enrolled, lost to follow-up and graduating is presented in Fig. 1. Demographic characteristics of programme graduates are presented in Table 2. Most participants were black (93·1 %), female (71·6 %) and aged 40 years or older (91·9 %). A majority of participants (65·3 %) received public health insurance and 86·6 % had a household income of less than $25 000 annually. Most were retired (24·3 %) and/or receiving disability benefits (40·2 %). At baseline, 60·4 % of participants were characterised as having low or very low food security and 59·4 % received Supplemental Nutrition Assistance Program benefits.

**Table 2:** Demographic characteristics of Georgia Food for Health programme graduates, 2017–2019 (*n* 282)

Characteristic	%
Sex	
Female	71·6
Male	28·4
Age in years	
18–29	2·6
30–39	5·5
40–49	17·6
50–59	42·1
60+	32·2
Race and ethnicity	
Black	93·1
White	1·5
Hispanic	2·2
Other/multi-racial	3·3
Highest level of education attained	
Less than high school degree	13·1
High school diploma or GED	35·3
Some college, did not graduate	27·6
College degree	13·8
Greater than college degree	10·2
Health insurance	
Not insured	20·9
Public insurance	65·3
Insured through employer	2·9
Private insurance	2·5
Other	8·3
Yearly income	
<25k	86·6
25–35k	6·9
>35k	6·5
Employment status	
Not employed	10·9
On disability	40·2
Working full-time	6·9
Working part-time	11·6
Retired	24·3
Student	1·1
Other	5·1
Food security status	
High or marginal	39·6
Low	41·9
Very low	18·5
SNAP participation	59·4

SNAP, Supplemental Nutritional Assistance Programme

Programme graduates are defined as those who completed at least 4 of the 6 monthly programme visits.

Food security status was assessed using the 6-item United States Department of Agriculture Household Food Security Survey Module and categorised as high or marginal (0–1 affirmative responses to screening questions), low (2–4 affirmative responses) or very low (5–6 affirmative responses).

Results of an attrition analysis show those retained in the programme (*n* 282) were more likely to be over the age of 50 years (*P* = 0·002) and less likely to have been referred from the infectious disease clinic (*P* = 0·023) compared to those lost to follow-up (*n* 49). No differences in retention were observed based on sex, race and ethnicity, highest level of education attained, employment, household income, household size, receipt of public benefits, health insurance or food security status at baseline.

### Clinical outcomes

At baseline, programme graduates had a mean BMI of 36·5 (95 % CI 35·5, 37·6) kg/m^2^, a mean weight of 227 (95 % CI 220, 233) lbs., a mean waist circumference of 45·3 (95 % CI 44·5, 46·1) inches, mean systolic blood pressure of 140·4 (95 % CI 138·1, 142·6) mmHg and mean diastolic blood pressure of 82·8 (95 % CI 80·4, 83·2) mmHg. In unadjusted models, we observed significant reductions in mean BMI, weight, waist circumference, systolic and diastolic blood pressure from the first programme visit attended to the last programme visit attended (Table 3).

**Table 3:** Unadjusted mean changes in clinical indicators between first and last visit attended, among Georgia Food for Health programme graduates, 2017–2019

Indicator	*n*	Mean at first visit	95 % CI	Mean at last visit	95 % CI	Mean difference	95 % CI	*t* Test *P*-value
BMI (kg/m^2^)	281	36·5	35·4, 37·5	36·2	35·2, 37·2	−0·3	–0·5, –0·0	0·02
Weight (lbs)	281	226·4	219·7, 233·1	224·8	218·0, 231·5	−1·6	–3·0, –0·2	0·03
Waist circumference (inches)	281	44·9	44·1, 45·8	43·4	42·6, 44·3	−1·5	–1·9, –1·1	<0·001
Systolic blood pressure (mmHg)	280	141·0	138·4, 143·5	135·8	133·7, 137·9	−5·2	–7·6, –2·8	<0·001
Diastolic blood pressure (mmHg)	280	82·2	80·7, 83·7	79·7	78·3, 81·0	−2·6	–4·0, –1·2	<0·001

After controlling for programme site, year of implementation, participant sex, race and ethnicity, Supplemental Nutrition Assistance Program status and household size, each additional programme visit beyond four visits was associated with a 0·6 (95 % CI –0·1, –0·0) kg/m^2^ reduction in BMI, a 0·4 (95 % CI –0·7, 0·0) lb. reduction in weight, a 0·4 (95 % CI –0·5, –0·3) inch reduction in waist circumference, a 1·0 (95 % CI –1·5, –0·6) mmHg reduction in systolic blood pressure and a 0·4 (95 % CI –0·7, –0·2) mmHg reduction in diastolic blood pressure (Table 4). Estimates using imputed data were consistent with those from the original dataset. However, coefficients for the association between programme participation and blood pressure were slightly lower in magnitude when using the imputed data (online Supplemental Table 1).

**Table 4:** Estimated association of an increase from 4 to 5 sessions and 5 to 6 sessions attended with change in clinical measures among Georgia Food for Health programme graduates, 2017–2019

Measure	N_participants_	Nobs	Mean obs per participant	Baseline mean	95 % CI	Unadjusted model	95 % CI	Adjusted model*	95 % CI	Adjusted model *P*-value
BMI (kg/m^2^)	262	1409	5·4	36·80	33·88, 39·72	−0·06	–0·11, 0·00	−0·06	–0·12, –0·01	0·024
Weight (lbs)	262	1409	5·5	227·66	213·29, 242·02	−0·31	–0·68, 0·05	−0·36	–0·74, 0·02	0·064
Waist circumference (inches)	262	1406	5·5	45·10	43·16, 47·05	−0·36	–0·47, –0·25	−0·37	–0·48, –0·27	<0·001
Systolic blood pressure (mmHg)	262	1406	5·5	139·79	137·33, 142·24	−0·97	–1·39, –0·55	−1·01	–1·45, –0·57	<0·001
Diastolic blood pressure (mmHg)	262	1406	5·3	81·73	78·50, 84·96	−0·42	–0·67, –0·17	−0·43	–0·69, –0·17	0·001

All estimates produced from linear mixed models including random intercepts and slopes for participants and site of participation.

* Adjusted models include fixed effects: year, sex, and age, race and ethnicity, Supplemental Nutrition Assistance Program participation status and household size

## Discussion

Among graduates of the GF4H programme, the number of programme visits attended was associated with modest but statistically significant reductions in BMI, weight, waist circumference and blood pressure measures. Most published studies on evaluations of similar programmes report increases in fruit and vegetable consumption and improvements in food security but have not reported on health outcomes^(16)^. A meta-analysis pooling results of three studies reporting BMI, four studies reporting blood pressure and five studies reporting HbA1c estimated that PRx programmes were associated with modest decreases in BMI by 0·6 kg/m^2^ (95 % CI –2·8, –0·3) and HbA1c by 0·8 % (95 % CI –1·6, –0·1) across studies^(16)^. In this meta-analysis, no significant changes in blood pressure or lipid concentrations were observed. Our results are generally comparable in magnitude to these few published evaluations of PRx programmes. However, heterogeneity in programme duration and implementation, participant characteristics and study design limit the ability to make direct comparisons between programmes. Other programmes range in duration from 13 weeks^(39,40)^ to 10 months^(41)^ and involve a variety of programme components such as mindfulness meditation and physical activity^(22)^.

Nutrition education components vary substantially across publications, with one programme providing healthy eating information handouts^(42)^, others involving one-on-one nutrition counselling sessions^(40,43)^ and another providing hour-long group-based sessions over a meal^(22)^. Although many programmes incorporate recipes and cooking demonstrations^(39,43)^, there are no published studies of PRx programmes that include hands-on cooking education. One study evaluating Cooking Matters, the evidence-based programme used in GF4H, demonstrated effectiveness in improving confidence with food resource management and food resource management practices such as comparison shopping and planning meals ahead of time^(44)^. In another study evaluating Cooking Matters in conjunction with Diabetes Self-Management Education and weekly meal provision (4 servings per week), improvements in diabetes management, diet, food security and health-related quality of life were observed^(45)^. No studies to our knowledge have evaluated health outcomes among participants of Cooking Matters, with the exception of Williams *et al.*, who reported no overall change in HbA1c in their study although participants experiencing food insecurity showed greater improvements in HbA1c than their food secure counterparts. While the monthly registered dietitian-led sessions, Eat Well, Live Well, included in the GF4H programme were not based on an existing evidence-based programme, the content included frequency and duration of sessions, and expertise of educators aligned with recognised best practices in nutrition education for low-income audiences^(46)^.

PRx programmes are designed to improve chronic disease risk factors by increasing food security and diet quality^(15)^. The combination of increased access to high-quality food and nutrition education supports participants’ engagement in healthy shopping and eating practices throughout the programme^(15,17,47)^. By practicing these behaviours, participants gain confidence in their skills and ability to acquire and cook healthy food on a budget, improving ability to maintain these behaviours after the programme has ended^(44,48)^. Sustained improvements in diet quality reduce the risk of chronic disease risk factor progression and exacerbation of existing conditions^(3)^. While evidence is converging to support the effectiveness of PRx programmes in improving food security, diet and self-efficacy related outcomes, results from studies reporting on health outcomes remain mixed. This study of the GF4H programme examining the association between programme attendance and health outcomes adds to the evidence of effectiveness of PRx on improving health risk factors and improves the literature base by including a majority black participant population, which is much-needed given health disparities evident among this group. Still, further studies are needed to examine the long-term benefits of these programmes and to better understand the impacts of individual programme components.

### Limitations

This study has several limitations. Follow-up data were not available for those who were lost to follow-up, limiting our findings to those who completed the programme. The mean number of visits attended for those lost to follow-up was 1·1 (95 % CI 1·0, 1·2), limiting our ability to investigate changes in the interim points for those who did not graduate. However, graduation rates across the 3 years of the programme were relatively high at 83 %, comparable to those observed in published evaluations of similar programmes^(22,39,42)^. For some clinics, participants were recruited from a pool of patients who had completed four introductory group nutrition classes, so those enrolled may have differed from the general patient population in that they may have been more motivated to participate based on previous positive experiences with the introductory programme or greater interest in diet-related programming. These participants may have also had more schedule flexibility to participate in the 6-month programme involving both group education sessions and weekly market visits for produce voucher redemption. It is also possible that those who graduated the programme remained engaged due to their perceived benefits of participation, indicating potential for reverse causality. However, the findings from this study remain useful for understanding the potential among motivated patients for chronic disease risk factor improvement after participation in a PRx programme.

We do not have information on why participants dropped out of the programme or were lost to follow-up. As Stotz and colleagues note, PRx participants often have competing barriers to programme engagement and may require additional services such as transportation to facilitate engagement^(49)^. Implementation of a process for routinely collecting and recording information on factors contributing to disengagement would be helpful for understanding the barriers to participation and generating ideas on how to address them to better retain participants.

Another limitation is the lack of a comparison group in evaluation. It is possible that changes observed in this study were related to factors outside of the intervention such as participation in other nutrition programming or factors tangential to effects of the intervention related to potential increases in engagement in care or improvements in medication adherence related to increases in food security. Additional investigations involving control groups and randomised study design are needed to strengthen our understanding of the potential of PRx programmes for achieving health outcome improvements.

Additionally, some missing data were present due to skipped questions in surveys or, in some cases, participants missing data collection days. While the proportion of missing data was low, analysis of a dataset created using multiple imputation was performed and compared to the results of complete-case analysis. Estimates of clinical change over the course of the programme were similar and help to confirm the validity of the findings presented here.

This study is also limited by the lack of ability to assess comparative effectiveness of the components of the programme on health outcomes. Although this study examined the relationship between programme attendance and CVD risk factors, it did not isolate the effects of nutrition education, cooking education and the provision of free produce. Future research to address this gap could involve the use of randomisation of programme components to allow for comparison. Additionally, structural equation modelling techniques such as pathway analysis could be useful for understanding the specific contributions of each component on different outcomes and help understand the role of mediating factors.

### Strengths

The major strengths of this evaluation include the use of 3 years of programme data from multiple sites of implementation and longitudinal data with objective biometric measures. This programme was implemented in an urban, safety net health system context, with low-income participants. These populations face the highest barriers to engaging with an in-person programme. However, we observed high graduation rates (83·0 % graduated across all years) and graduation improved with each year of programme implementation (from 79·1 % in 2017 and 2018 to 90·8 % in 2019). Improvements in programme graduation are potentially related to continuity of staff and increased competence with operating procedures over time including increased communication between programme partners, resulting in greater clarity of goals and a more cohesive and flexible programme structure for participants^(50)^.

## Conclusions

Overall, our findings support the hypothesis that increased access to fresh produce and education in nutrition, cooking and food resource management techniques is associated with modest improvements in chronic disease risk factors over the course of a 6-month intervention in a low-income, urban population. Each additional programme visit attended beyond the graduation threshold was associated with modest but significant improvements in CVD risk factors, suggesting that increased engagement in cooking and nutrition education within the context of a PRx programme improves health outcomes. These findings can also help with participant and programme staff goal setting and inform realistic outcomes from participation in similar programmes.

## Supplementary material

For supplementary material accompanying this paper visit https://doi.org/10.1017/S1368980023001611. click here to view supplementary material
