# Supplementary material for: Participation in the Georgia Food for Health programme and CVD risk factors: a longitudinal observational study
Source: Public Health Nutr. 2023 Aug 7;26(11):2470–9. doi: 10.1017/S1368980023001611 (PMC10641606; doi:10.1017/S1368980023001611)
Supplement: Supplementary file 1 [file S1368980023001611sup001.docx]

**Supplemental Table 1.** Estimated association of total monthly program visits attended (1-6) with change in clinical measures after multiple imputation among Georgia Food for Health (GF4H) program graduates, 2017-2019

| **Measure** | **N_participants_** | **N_Obs_** | **Mean obs per participant** | **Baseline Mean**  **(95% CI)** | **Unadjusted Model (95% CI)** | **Model 2*** |
| --- | --- | --- | --- | --- | --- | --- |
| BMI (kg/m2) | 282 | 1,510 | 5.4 | 36.79  (33.88, 39.69) | -0.06  (-0.11, -0.00) | -0.06  (-0.11, -0.00) |
| Weight (lbs) | 282 | 1,513 | 5.4 | 227.52  (213.30, 241.74) | -0.33  (-0.70, 0.04) | -0.36  (-0.74, 0.01) |
| Waist circumference (inches) | 282 | 1,513 | 5.4 | 45.10  (43.16, 47.03) | -0.36  (-0.47, -0.25) | -0.36  (-0.47, -0.27) |
| Systolic blood pressure (mmHg) | 282 | 1,513 | 5.4 | 139.63  (137.17, 142.10) | -0.94  (-1.36, -0.52) | -0.95  (-1.37, -0.54) |
| Diastolic blood pressure (mmHg) | 282 | 1,513 | 5.4 | 81.64  (78.43, 84.85) | -0.40  (-0.66, -0.15) | -0.41  (-0.65, -0.15) |

All estimates produced from linear mixed models including random intercepts and slopes for participants and site of participation

*Adjusted models include fixed effects: year, sex, age, race & ethnicity, and supplemental nutrition assistance program (SNAP) participation, & household size
